# Theoretical Implications of a Pre-Erythrocytic *Plasmodium vivax* Vaccine for Preventing Relapses

**DOI:** 10.1016/j.pt.2016.12.011

**Published:** 2017-04

**Authors:** Michael White, Rogerio Amino, Ivo Mueller

**Affiliations:** 1 MRC Centre for Outbreak Analysis & Modelling, Department of Infectious Disease Epidemiology, Imperial College London, UK; 2 Division of Population Health & Immunity, Walter and Eliza Hall Institute, Melbourne, Australia; 3 Department of Parasites & Insect Vectors, Institut Pasteur, Paris, France

**Keywords:** *Plasmodium vivax*, malaria, vaccine, relapse, hypnozoite

## Abstract

Preventing malaria infection through vaccination requires preventing every sporozoite inoculated by mosquito bite: a major challenge for *Plasmodium falciparum*. *Plasmodium vivax* sporozoites consist of tachysporozoites causing primary infection and bradysporozoites leading to relapses. We hypothesise that a candidate *P. vivax* vaccine with low efficacy against primary infection may substantially reduce transmission by preventing relapses.

Considerable effort has been invested in the development of pre-erythrocytic vaccines for the prevention of *P. falciparum* infection, with several candidates demonstrating significant efficacy against infection in controlled human malaria infection (CHMI) studies [1]. One candidate vaccine, RTS,S/AS01, has recently completed phase 3 trials in young children in sub-Saharan Africa [2], receiving a positive opinion from the European Medicines Agency and recommended for pilot implementation studies by the World Health Organization. In the month following vaccination, RTS,S/AS01 prevents approximately 50% of infections in malaria-naïve adults by mounting humoral and cellular immune responses targeting the circumsporozoite (CS) antigen [1]. Protection is strongly associated with vaccine-induced anti-CS IgG antibodies [3] which are believed to immobilise sporozoites, preventing their invasion from the skin into blood vessels [4]. Based on the delayed time to detectable blood-stage parasitemia in vaccinated volunteers who were not protected, it has been estimated that RTS,S/AS01 immobilises 90–95% of sporozoites, with the 5–10% of breakthrough sporozoites being sufficient to allow infection half of the time [3].

Similarly to *P. falciparum*, infection with *P. vivax* begins with the inoculation of sporozoites from an infectious mosquito. *P. vivax* sporozoites can be categorised into tachysporozoites which immediately develop into exoerythrocytic schizonts, and bradysporozoites which develop into hypnozoites where development is arrested for weeks to years until activation to cause relapses [5]. An antibody-mediated pre-erythrocytic *P. vivax* vaccine is likely to target both tachysporozoites and bradysporozoites in the skin. Similarly to *P. falciparum*, a single tachysporozoite that evades the vaccine-induced immune response will lead to a breakthrough infection, thus we would expect low to modest efficacy against primary infection even for a vaccine that prevents a large proportion of sporozoites. However, it is possible that every bradysporozoite immobilised may lead to a relapse prevented, thus efficacy against relapses may be comparable to the proportion of sporozoites prevented. This hypothesis is illustrated further via the example in Figure 1.

The contribution of relapses to sustaining *P. vivax* transmission has been investigated in epidemiological studies, with studies incorporating treatment with primaquine for the elimination of hypnozoites from the liver providing a particularly rich source of information. In treatment-reinfection studies in southeast Asian and western Pacific populations exposed to *P. vivax*, where participants were randomised to receive blood-stage drugs (e.g., chloroquine or artemether-lumefantrine) or blood-stage drugs plus primaquine, it has been demonstrated that approximately 80–90% of new infections are attributable to relapses 6, 7. This suggests that interventions that target hypnozoites, either by elimination from the liver through treatment or preventing their development in the first place, may cause substantial reductions in population-level transmission of *P. vivax*.

The theoretical implications of using vaccination to inhibit the build-up of the hypnozoite reservoir in a population were investigated using a mathematical model of within-host hypnozoite infection coupled to a model of *P. vivax* transmission between humans and mosquitoes [8]. We consider a hypothetical nonwaning pre-erythrocytic *P. vivax* vaccine with efficacy against primary infection of *V*_inf_ and efficacy against relapses of *V*_rel_. Figure 2A shows the effect of vaccination on the individual-level in terms of the expected reduction in time with blood-stage parasitemia. The duration of blood-stage parasitemia is a proxy for the onwards transmission potential to mosquitoes because of the high degree of correlation between *P. vivax* parasitemia and gametocytemia [9]. As approximately 80% of infections are assumed to be attributable to relapses, increasing *V*_rel_ is predicted to cause large reductions in the time spent with blood-stage parasitemia. The population-level impact of a vaccination campaign with 70% coverage is shown in Figure 2B for a range of scenarios. A vaccine that does not prevent relapses is predicted to cause very limited reductions in *P. vivax* parasite prevalence. In contrast, a vaccine with high efficacy against relapses is predicted to cause substantial reductions in prevalence, enough to interrupt transmission in the simplified scenario represented here.

The potentially high efficacy against relapses of a vaccine with low to modest efficacy against primary infection, coupled with the crucial role of relapses in sustaining transmission, suggests that pre-erythrocytic *P. vivax* vaccines be prioritised for further investigation. However, a number of factors may count against the outlined hypothesis, primarily related to knowledge gaps surrounding hypnozoites and relapses. The factors governing commitment of sporozoites to development as schizonts or hypnozoites are not understood. The precise numbers of tachysporozoites and bradysporozoites are not known, although it has been demonstrated that the ratio of schizonts to hypnozoites is strain-dependent [10]. In a prescient but largely ignored analysis, a group of Russian malariologists demonstrated that the expected number of relapses is dependent on the ratio of tachysporozoites to bradysporozoites [5]. Furthermore, the processes governing activation of hypnozoites to initiate relapses are still poorly understood. If hypnozoites activate independently (e.g., without external triggers or quorum sensing) this would suggest one relapse prevented for every one hypnozoite prevented. External triggers such as fevers may lead to hypnozoite activation in batches – in which case prevention of relapse requires preventing every hypnozoite in the batch.

In addition to the limitations of our understanding of hypnozoites, there is considerable uncertainty of the mechanism of action of pre-erythrocytic vaccines against any *Plasmodium* parasite. Whereas antibody-mediated responses are likely to target sporozoites in the skin, cell-mediated immune responses are more likely to target liver-stage schizonts. A vaccine-induced cell-mediated response may not be able to target a hypnozoite until after activation – potentially greater than a year after the initial infectious mosquito bite, and long enough for significant waning of vaccine-induced immunity [1].

In participants in malaria-therapy studies reinfected with a homologous strain of *P. vivax*, large reductions in blood-stage parasite densities between primary and secondary infections were observed [11]. Given the correlation between parasitemia and gametocytemia [9], this suggests that a primary infection may contribute proportionately more to onwards transmission than a single relapse. As such, there is a strong case for the incorporation of additional blood-stage antigens in a candidate vaccine to reduce parasitemia and gametocytemia in breakthrough infections. Acknowledging the limitations of our understanding of the biology and vaccinology, past experience of developing *P. falciparum* vaccines with low to moderate efficacy against infection suggests that it is also feasible to do so for *P. vivax* and subsequently investigate the impact on relapses. However, there are few *P. vivax* vaccine candidates currently under development, based on a limited number of antigens, with most projects still at a preclinical phase [12]. It is important that underinvestment doesn’t allow the potential of *P. vivax* vaccines to continue to be overlooked.

## Figures and Tables

**Figure 1:**
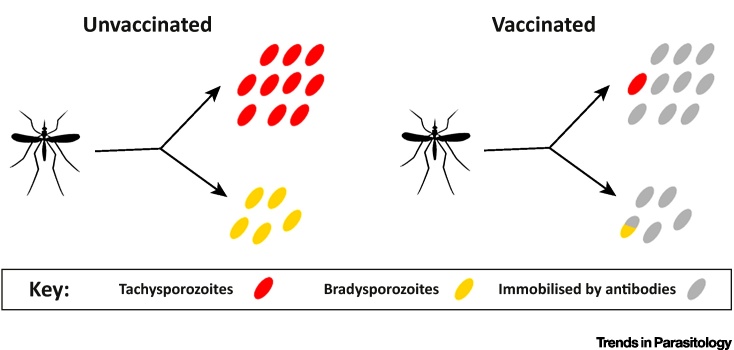
Example of a Bite from a *Plasmodium vivax* Infectious Mosquito Inoculating 15 Sporozoites Consisting of 10 Tachysporozoites That Immediately Develop into Exoerythrocytic Schizonts, and 5 Bradysporozoites That Develop into Hypnozoites.--> In an unvaccinated individual, the 10 tachysporozoites will cause a single primary blood-stage infection, and the hypnozoites will cause up to five relapses. Some hypnozoites may die within liver hepatocytes or activate in batches, giving rise to fewer than five relapses. In an individual receiving a vaccine that immobilises 90% of sporozoites, the efficacy against primary infection will be 0.9^10^ = 35%. However, every hypnozoite immobilised may potentially lead to a relapse prevented, and consequently an efficacy against relapses of up to 90%.

**Figure 2:**
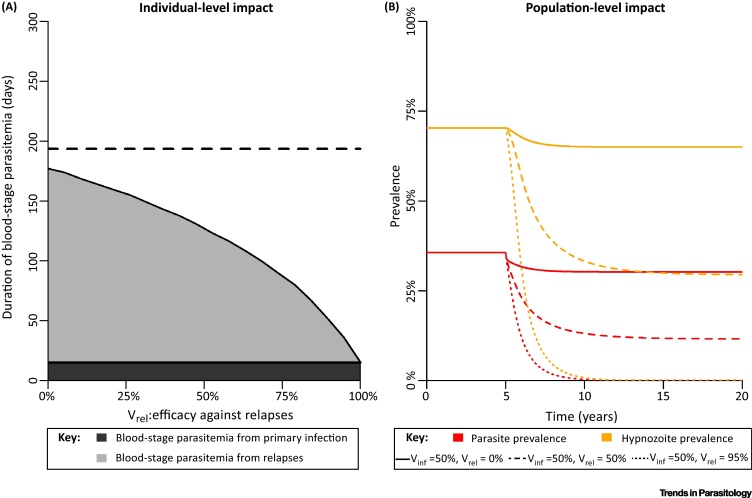
Simulations from a Mathematical Model of the Impact of a Hypothetical Nonwaning Pre-Erythrocytic *Plasmodium vivax* Vaccine. (A) By preventing infection, a vaccine can reduce the expected duration of blood-stage parasitemia. Efficacy against primary infection of *V*_inf_ = 50% and duration of each blood-stage infection (primary or relapse) of 1 month are assumed. The dashed line indicates the expected duration of blood-stage parasitemia in an unvaccinated individual. (B) Predicted impact of a vaccination campaign with 70% coverage on population-level transmission. The R computer code for generating this figure is provided in the, supplemental information online allowing for simulation with different parameter values.

## References

[1] Kester K.E. (2009). randomized, double-blind, phase 2a trial of falciparum malaria vaccines RTS,S/AS01B and RTS,S/AS02A in malaria-naive adults: safety, efficacy, and immunologic associates of protection. J. Infect. Dis..

[2] Tinto H. (2015). Efficacy and safety of RTS,S/AS01 malaria vaccine with or without a booster dose in infants and children in Africa: final results of a phase 3, individually randomised, controlled trial. Lancet.

[3] White M.T. (2013). The relationship between RTS,s vaccine-induced antibodies, CD4(+) T cell responses and protection against *Plasmodium falciparum* infection. PLoS One.

[4] Vanderberg J.P., Frevert U. (2004). Intravital microscopy demonstrating antibody-mediated immobilisation of *Plasmodium berghei* sporozoites injected into skin by mosquitoes. Int. J. Parasitol..

[5] Lysenko A.J. (1977). Population studies of *Plasmodium vivax*. 1. The theory of polymorphism of sporozoites and epidemiological phenomena of tertian malaria. Bull. World Health Organ..

[6] Luxemburger C. (1999). Treatment of *vivax* malaria on the western border of Thailand. Trans. Roy. Soc. Trop. Med. Hyg..

[7] Robinson L.J. (2015). Strategies for understanding and reducing the *Plasmodium vivax* and *Plasmodium ovale* hypnozoite reservoir in Papua New Guinean children: a randomised placebo-controlled trial and mathematical model. PLoS Med..

[8] White M.T. (2014). Modelling the contribution of the hypnozoite reservoir to *Plasmodium vivax* transmission. Elife.

[9] Koepfli C. (2015). Blood-stage parasitaemia and age determine *Plasmodium falciparum* and *P. vivax* gametocytaemia in Papua New Guinea. PLoS One.

[10] Mikolajczak S.A. (2015). *Plasmodium vivax* liver stage development and hypnozoite persistence in human liver-chimeric mice. Cell Host Microbe.

[11] Collins W.E. (2004). A retrospective examination of reinfection of humans with *Plasmodium vivax*. Am. J. Trop. Med. Hyg..

[12] Mueller I. (2015). Development of vaccines for *Plasmodium vivax* malaria. Vaccine.

